# Oxidative stress-related circulating miRNA-27a is a potential biomarker for diagnosis and prognosis in patients with sepsis

**DOI:** 10.1186/s12865-022-00489-1

**Published:** 2022-03-25

**Authors:** Yingwei Ou, Rongcheng An, Haochu Wang, Lue Chen, Yong Shen, Wenwei Cai, Wei Zhu

**Affiliations:** 1 Department of Emergency, Zhejiang Provincial People’s Hospital (Affiliated People’s Hospital, Hangzhou Medical College), 158 Shangtang Road, Hangzhou, 310014 Zhejiang People’s Republic of China; 2 Department of Radiology, Zhejiang Provincial People’s Hospital (Affiliated People’s Hospital, Hangzhou Medical College), 158 Shangtang Road, Hangzhou, 310014 Zhejiang People’s Republic of China

**Keywords:** Sepsis, microRNA-27a, Oxidative stress, Diagnosis, Prognosis

## Abstract

**Background:**

Oxidative stress plays a critical role on the processes of sepsis, and several microRNAs have been identified that may regulate the occurrence of oxidative stress. However, the relation between oxidative stress-related microRNA 27a (miR-27a) and sepsis is unknown. The present study aimed to determine the value of circulating miR-27a for the diagnosis and prognosis of sepsis.

**Methods:**

This retrospective study included 23 patients with sepsis and 25 without sepsis treated at the emergency intensive care unit (EICU) or our institution between January 2019 and January 2020. Levels of circulating miR-27a and levels of oxidative stress-related indicators were measured and compared between sepsis and non-sepsis patients. Receiver operating characteristic (ROC) curve analysis was used to determine diagnostic efficiency of miR-27a.

**Results:**

Circulating miR-27a levels in sepsis patients were higher than those in non-sepsis patients (p < 0.05), and levels were significantly higher in patients that died than those that lived (p < 0.05). In patients with sepsis, circulating miR-27a level was positively correlated with serum malondialdehyde (MDA) level (rs = 0.529, p = 0.007), and negatively correlated with serum glutathione peroxidase (GSH-Px) level (rs = − 0.477, p = 0.016). No significant correlation was observed between circulating miR-27a and serum superoxide dismutase (SOD) in sepsis patients (rs = − 0.340, p = 0.096). The area under the ROC curve (AUC) of miR-27a level for prediction of sepsis was 0.717 (p = 0.009) and for 28-day mortality was 0.739 (p = 0.003).

**Conclusions:**

This study showed that circulating miR-27a level is correlated with oxidative stress and mortality in patients with sepsis, and may serve as a potential non-invasive molecular biomarker.

## Background

Sepsis, also referred to as severe systemic inflammatory disease, is a lethal response of the host to microbial infection, and its high mortality is related to an imbalance of inflammatory mediators [1]. Sepsis is commonly due to Gram-negative bacteria, and can lead to multiple organ dysfunctions [2–5]. Although great progress has been made in the understanding and treatment of sepsis and its complications, the mortality rate associated with sepsis has not improved [5–7]. In a retrospective cohort study of sepsis patients seen in the emergency department [ED], 61 of 98 patients (62%) had sepsis-related death [5].

Biomarkers have been suggested as a method to assist in the early diagnosis of sepsis, and thus initiate appropriate treatment early [8]. Biomarkers are defined as biological molecules that are objectively measured as indicators of normal biological processes, pathogenic processes, or pharmacological responses to therapeutic interventions [9]. The ideal sepsis biomarker should be easy and inexpensive to measure, easy to obtain, be highly specific and sensitive for a diagnosis of sepsis, and be useful for monitor the disease course and response to treatment. Although 178 biomarkers have been described in the literature, many are not used in clinical practice because of their lack of sensitivity and specificity [10], and no single biomarker with sufficient sensitivity and specificity has been identified [10, 11]. New categories of biomarkers, such as microRNAs (miRNAs), have drawn considerable attention a diagnostic and prognostic markers of sepsis.

miRNAs do not encode proteins, but regulate gene expression by inhibiting the translation or transcription of its target mRNA. Recent study has reported that miRNAs are released into circulation, and that circulating miRNA profiles may change under various pathological conditions, such as inflammation, infection, and sepsis [12]. Using array-based and single PCR-based methods, various dysregulated miRNAs have been described in the context of sepsis including miR-25, miR-133a, miR-146, miR-150, and miR-223 [12]. Some miRNAs have been shown to be related to disease stage, and short-term and long-term prognosis of patients with sepsis [12]. Because of the close relations between certain miRNAs and the prognosis of patients with sepsis, a number of miRNAs have been examined for their potential as biomarkers of sepsis [13–16].

In a study using a murine model of septic liver injury, the level of miR-27a from liver tissues was associated with the level of oxidative stress [17], indicating the miR-27a can reflect oxidative stress in vivo. Prior study has also shown that miR-27a levels are increased in the lungs of septic mice [18]. Knocking down miR-27a down-regulates the expression levels of tumor necrosis factor α (TNF-α) and interleukin-6 (IL-6) by reducing the phosphorylation of nuclear factor-κB (NF- κB) and inhibiting its DNA binding activity [18]. Neutralization of miR-27a up-regulates peroxisome proliferator-activated receptor γ (PPARγ), relieves pulmonary inflammation, and promotes the survival of septic mice [18]. Taken together, the aforementioned studies suggest miR-27a plays an important role in regulating the oxidative stress-related inflammatory response in sepsis. However, few studies have investigated the association of circulating miR-27a levels with oxidative stress-related sepsis in humans, and no study has determined a cutoff value of circulating miR-27a to predict sepsis and sepsis-related mortality in humans. Therefore, we hypothesized that oxidative stress-related miR-27a may contribute to development of sepsis and be a potential biomarker for the diagnosis and prognosis of sepsis.

## Results

### Patient demographics and clinical characteristics

The baseline characteristics of the 23 sepsis patients and 25 non-sepsis patients are shown in Table 1. Age, sex distribution, and comorbidities were not different between the 2 groups (all, p > 0.05). The median age of patients with sepsis was 65 years (range: 40–92 years), 56.5% were male, 30.4% had hypertension, and 21.7% had diabetes. The median age of non-sepsis patients was 58 years (range: 37–91 years), 56.0% were male, 36.0% had hypertension, 4.0% had cardiovascular disease (CVD), and 20.0% had diabetes.

**Table 1:** Demographic and clinical characteristics of patients

	Total (N = 48)	Non-sepsis (n = 25)	Sepsis (n = 23)	p-value
Age (years), median (range)	65 (37–92)	58 (37–91)	65 (40–92)	0.200
Sex, n (%)				1.000
Male	27 (56.3%)	14 (56.0%)	13 (56.5%)	
Female	21 (43.8%)	11 (44.0%)	10 (43.5%)	
Hypertension, n (%)	16 (33.3%)	9 (36.0%)	7 (30.4%)	0.765
CVD, n (%)	1 (2.1%)	1 (4.0%)	0 (0.0%)	1.000
DM, n (%)	10 (20.8%)	5 (20.0%)	5 (21.7%)	1.000
APACHE II score, median (range)	22 (4–32)	20 (4–32)	25 (17–32)	0.017*
SOFA score, median (range)	8 (2–24)	4 (2–24)	12 (5–21)	< 0.001*
Infectious shock, n (%)	15 (31.3%)	0 (0.0%)	15 (65.2%)	< 0.001*
Multiple organ failure, n (%)	5 (10.4%)	0 (0.0%)	5 (21.7%)	0.020*
CRP (mg/L), median (range)	241.5 (4.9–365.9)	167.0 (4.9–365.9)	257.3 (36.4–355.8)	0.040*
PCT (ng/mL), median (range)	21.8 (0.1–100.0)	2.3 (0.1–85.0)	62.3 (3.9–100.0)	< 0.001*
WBC (× 10^9^/L)**,** median (range)	16.8 (2.3–61.7)	15.5 (4.7–37.3)	19.9 (2.3–61.7)	0.044*
MDA (nmol/mL), median (range)	29.4 (0.6–361.7)	11.8 (0.6–361.7)	51.8 (2.6–263.9)	0.011*
SOD (U/mL), median (range)	22.0 (0.3–118.9)	40.3 (0.3–118.9)	20.3 (3.3–101.2)	0.040*
GSH-Px (IU), median (range)	1583.0 (555.6–3787.8)	2350.4 (555.6–3737.4)	1479.2 (638.9–3787.8)	< 0.001*
MiR-27a, median (range)	1.0 (0.1–3.5)	0.7 (0.1–2.1)	1.2 (0.2–3.5)	0.010*
28-day mortality, n (%)	25 (52.1%)	9 (36.0%)	16 (69.6%)	0.025*
ICU stay, median (range)	9 (1–71)	13 (2–71)	8 (1–48)	0.230
Hospital stat, median (range)	16 (1–71)	19 (2–71)	13 (1–54)	0.160

CVD, cardiovascular disease; CRP, C-reactive protein; PCT, procalcitonin; WBC, white blood cell; APACHE II, Acute Physiology and Chronic Health Evaluation II; SOFA, Sequential Organ Failure Assessment; SOD, superoxide dismutase; MDA, Malondialdehyde; GSH-Px, glutathione peroxidase

*p < 0.05

Compared to the non-sepsis patients, sepsis patients had increased levels of PCT, CRP, and WBC count (all, p < 0.05). In addition, APACHE II score, SOFA score, the occurrence of multiple organ failure, and 28-day mortality were all significantly greater in sepsis patients (all, p < 0.05).

### Expression of miR-27a and levels of oxidative stress-related indicators

**Fig. 1:**
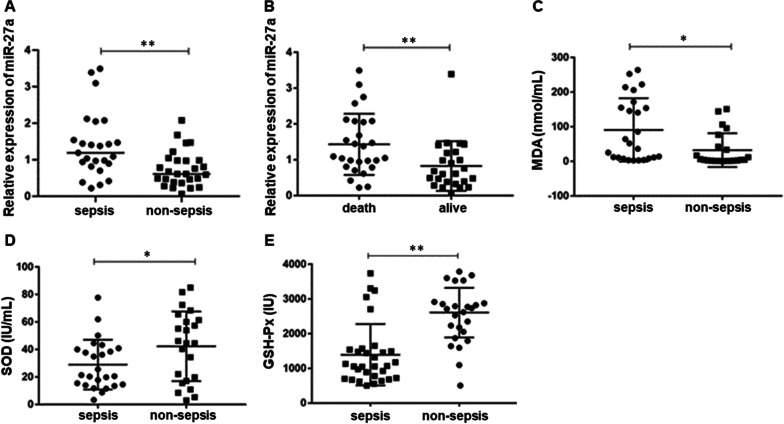
Expression of miR-27a and oxidative stress-related indicators. **A** miR-27a expression in sepsis and non-sepsis patients was detected by qPCR. **B** Patients were divided into those that died within 28 days and survived for more than 28 days, and the expression of microRNA 27a was compared between the 2 groups. **C**–**E** Indicators of oxidative stress were measured by ELISA, and the levels of these indicators were compared between sepsis and non-sepsis patients. *p < 0.05; **p < 0.01

The level of circulating miR-27a in patients with sepsis patients was higher than in patients without sepsis (1.2 (0.2–3.5) vs. 0.7 (0.1–2.1) ng/mL, respectively, p < 0.05 (Table 1 and Fig. 1A). We further determined whether circulating miR-27a level was associated with 28-day mortality. As shown in Table 2 and Fig. 1B, the circulating miR-27a level was significantly increased patients who died than those who remained alive (1.0 (0.2–3.5) vs. 0.8 (0.1–3.1) ng/mL, respectively, p < 0.05).

**Table 2:** Association of patient characteristics with 28-day mortality

	Total (N = 48)	Alive (n = 23)	Dead (n = 25)	p-value
Age (years), median (range)	65 (37–92)	58 (37–87)	67 (40–92)	0.059
Sex, n (%)				0.145
Male	27 (56.3%)	10 (43.5%)	17 (68.0%)	
Female	21 (43.8%)	13 (56.5%)	8 (32.0%)	
Hypertension, n (%)	16 (33.3%)	6 (26.1%)	10 (40.0%)	0.368
CVD, n (%)	1 (2.1%)	0 (0%)	1 (2.1%)	1.000
DM, n (%)	10 (20.8%)	3 (13.0%)	7 (28.0%)	0.292
APACHE II score, median (range)	22 (4–32)	21 (4–30)	25 (15–32)	0.015*
SOFA score, median (range)	8 (2–24)	4 (2–15)	10 (2–24)	< 0.001*
Infectious shock, n (%)	15 (31.3%)	4 (17.4%)	11 (44.0%)	0.065
Multiple organ failure, n (%)	5 (10.4%)	0 (0.0%)	5 (20.0%)	0.020*
CRP (mg/L), median (range)	241.5 (4.9–365.9)	176.9 (4.9–325.0)	259.3 (36.4–365.9)	0.062
PCT (ng/mL), median (range)	21.8 (0.1–100.0)	2.5 (0.1–100.0)	47.0 (1.0–100.0)	0.003*
WBC (× 10^9^/L)**,** median (range)	16.8 (2.3–61.7)	17.2 (10.4–54.2)	15.3 (2.3–61.7)	0.197
MDA (nmol/mL), median (range)	29.4 (0.6–361.7)	25.2 (0.6–361.7)	36.1 (1.2–252.4)	0.421
SOD (U/mL), median (range)	22 (0.3–118.9)	22.0 (0.3–105.7)	21.4 (3.3–118.9)	0.613
GSH-Px (IU), median (range)	1583.0 (555.6–3787.8)	1873.0 (555.6–3680.9)	1569.4 (722.2–3787.8)	0.710
MiR-27a, median (range)	1.0 (0.1–3.5)	0.8 (0.1–3.1)	1.0 (0.2–3.5)	0.013*
Sepsis, n (%)	23 (47.9%)	7 (30.4%)	16 (64.0%)	0.025*
ICU stay, median (range)	8 (1–48)	7 (2–48)	9 (1–41)	0.717
Hospital stat, median (range)	15 (1–59)	24 (5–59)	10 (1–41)	0.010*

CVD, cardiovascular disease; CRP, C-reactive protein; PCT, procalcitonin; WBC, white blood cell; APACHE II, Acute Physiology and Chronic Health Evaluation II; SOFA, Sequential Organ Failure Assessment; SOD, superoxide dismutase; MDA, Malondialdehyde; GSH-Px, glutathione peroxidase

*p < 0.05

As shown in Table 1 and Fig. 1C–E, MDA level was significantly increased in patients with sepsis compared to non-sepsis patients, while SOD and GSH-Px levels were significantly decreased in sepsis patients compared with non-sepsis patients (all, p < 0.05). However, the levels of MDA, SOD, and GSH-Px were not significantly associated with 28-day mortality (Table 2, all, p > 0.05).

### Correlations of miR-27a with oxidative stress indicators

**Fig. 2:**
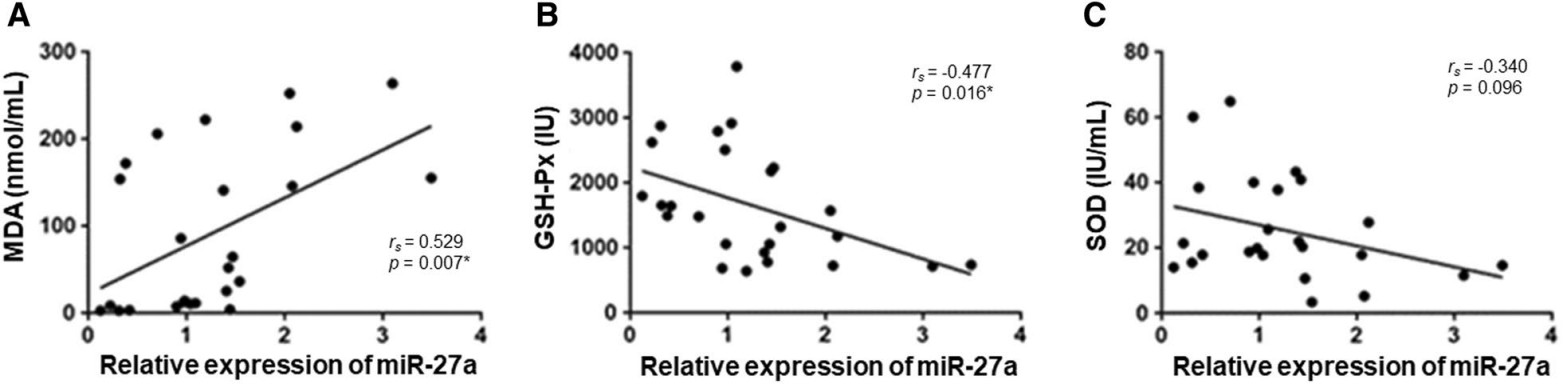
Correlation between microRNA 27a and oxidative stress. Spearman’s correlation test was performed to analyze the data. Circulating miR-27a was positively correlated with MDA (**A**) and GSH-Px (**B**). **C** The relation between miR-27a and SOD was not significant difference. *p < 0.05

Next, we determined whether circulating miR-27a level was associated with oxidative stress indicators in sepsis patients. As shown in Fig. 2A, B, circulating miR-27a level was positively correlated with MDA level (r_s_ = 0.529, p = 0.007) in sepsis patients, while it was negatively correlated with GSH-Px level (r_s_ = -0.477, p = 0.016) in sepsis patients. No significant correlation between circulating miR-27a and SOD was observed in patients with sepsis (r_s_ = − 0.340, p = 0.096) (Fig. 2C).

### Diagnostic and prognosis efficiency of miRNA-2a and other indices in sepsis

**Fig. 3:**
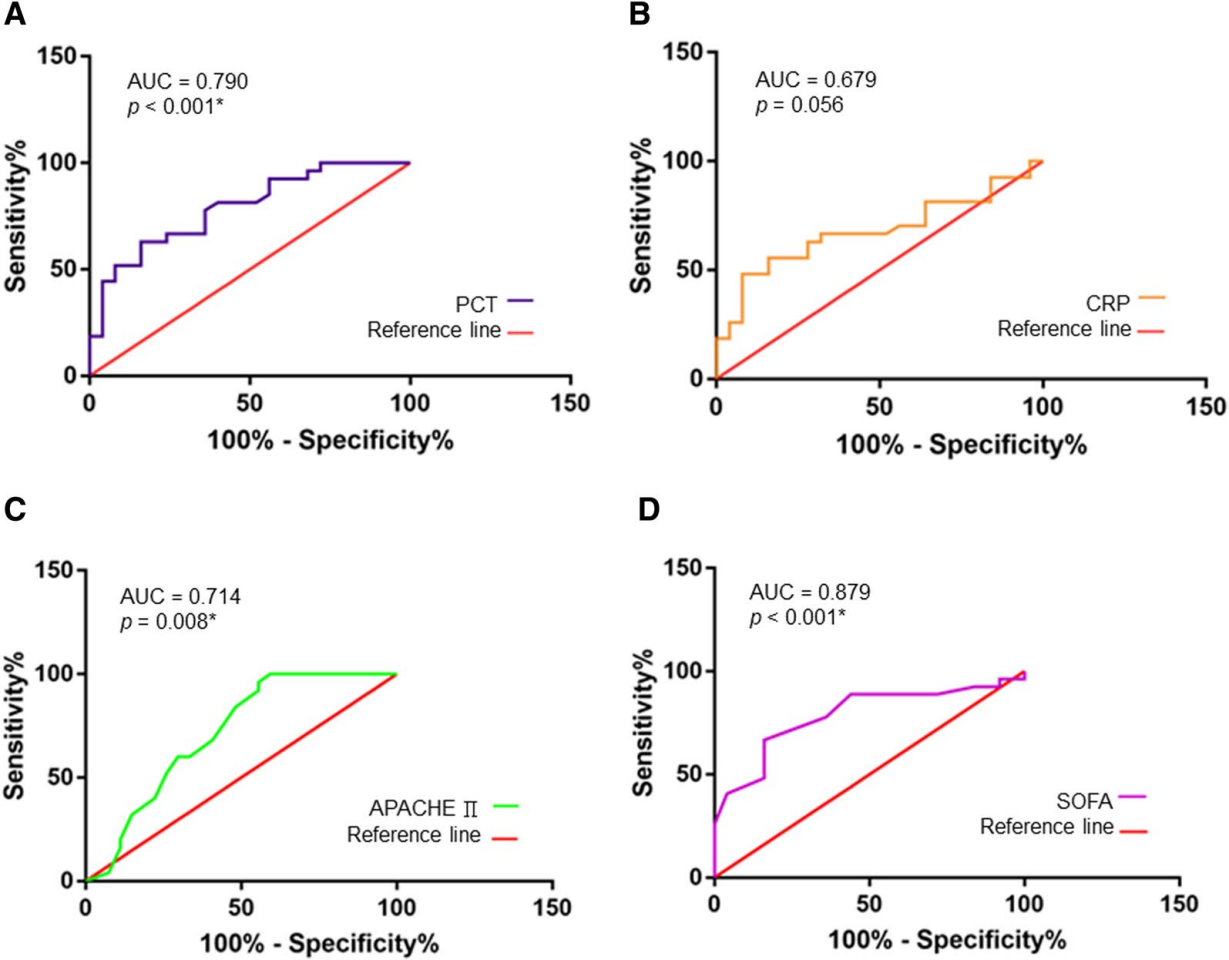
Receiver operating characteristic (ROC) curves of clinical parameters. Based on the clinical data collected, ROC analysis was performed for PCT levels (**A**), CRP levels (**B**), APACHE II score (**C**), and SOFA score (**D**) for diagnosis of sepsis. *p < 0.05

ROC curve analysis indicated that the area under the ROC curve (AUC) of PCT for a diagnosis of sepsis was 0.790 (p < 0.001, Fig. 3A) and of CRP was 0.679 (p = 0.056, Fig. 3B), although CRP did not reach statistical significance. ROC analysis of APACHE II score indicate and AUC of 0.714 (p = 0.008, Fig. 3C) for a diagnosis of sepsis, and the AUC for SOFA score was 0.879 (p < 0.001, Fig. 3D).

**Fig. 4:**
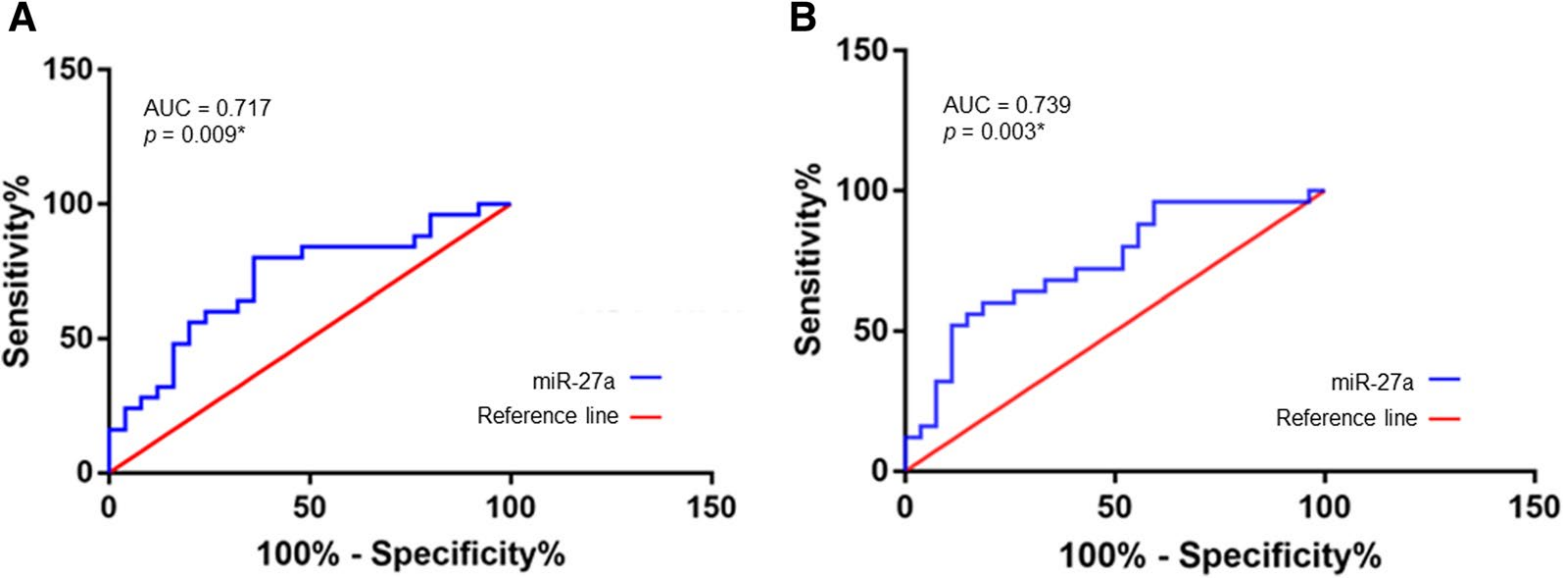
Receiver operating characteristic (ROC) curves of miR-27a. The diagnostic efficiency of miR-27a was determined by ROC curve analysis (**A**). ROC curve of the prognostic value for 28-day mortality of miR-27a (**B**). *p < 0.05

As shown in Fig. 4, the AUC of circulating miR-27a for diagnosis of sepsis was 0.717 (p = 0.009) (with 80% sensitivity and 64% specificity) and for predicting 28-day mortality was 0.739 (p = 0.003).

## Discussion

Sepsis and septic shock syndrome represent major issues in medicine. Although improvements have been made in the diagnosis and treatment of sepsis [1], in the same year the new guidelines were released Williams et al. [19] demonstrated in 2016 that the new diagnostic model is not very sensitive in the early stages of the disease. The current analysis of clinical parameters of sepsis and non-sepsis patients yielded the following new key findings. First, circulating miR-27a level was significantly increased in sepsis patients. Second, circulating miR-27a level was correlated to oxidative stress indicators (MDA and GSH-Px) in sepsis patients. Third, patients with higher circulating miR-27a levels or sepsis had high 28-day mortality. These findings suggest that miR-27a may be a potential biomarker for the diagnosis and prognosis of sepsis.

The pathogenesis of sepsis, a severe systematic inflammatory disease, is unusually complex. Oxidative stress, however, has been accepted to play an important role in the pathophysiology of sepsis [20–23]. Under normal physiologic conditions, redox balance exists through a complex interplay of genes that mediate oxidant generation and antioxidant responses. An imbalance between the production of reactive oxygen species (ROS) and the capacity for detoxification of their reactive intermediates results in oxidative stress. Similarly, the present study showed that oxidative stress indicators, including MDA, SOD, and GSH-Px, were significantly different between sepsis and non-sepsis patients. Interestingly, we further found that circulating miR-27a level was significantly associated with levels of MDA or GSH-Px in sepsis patients. Based on these findings, we speculate that circulating miR-27a-mediated sepsis may be associated with the molecular mechanisms of oxidative stress. Certainly, it merits further investigation.

The activation of oxidative stress causes the production of ROS, together with the recruitment of inflammatory cytokines [24], contributing to cellular mitochondrial dysfunction [25] and subsequent multi-organ failure [26]. Based on the function of oxygen free radicals in cellular signal transduction and gene activation [27], studies of the molecular mechanisms of oxidative stress in mitochondrial may provide a novel therapeutic approach for sepsis [28]. Previous experiments have confirmed that miR-27a has a role in both inflammation and mitochondrial function. In alveolar epithelial cells, knocking down miR-27a reduced expression of tumor necrosis factor alpha (TNF-α) and interleukin-1 (IL-6) under lipopolysaccharide (LPS) stimulation through the decreased phosphorylation of NF-κB p65 [18]. However, in mice with acute lung injury (ALI), overexpression of miR-27a by agomir-27a improved lung injury and ameliorated lung inflammation via decreasing TLR4/MyD88/NF-κB activation, evidenced by decreased levels of TNF-α, IL-6, IL-1β, and myeloperoxidase (MPO) activity in bronchoalveolar lavage fluid [29]. In the kidney tissue, inhibition of miR-27a-3p relieved mitochondrial dysfunction, manifesting as up-regulation of mitochondrial membrane potential, complex I and III activities, adenosine triphosphate and mitochondrial cytochrome C, and decreased production of ROS [30]. In tumor cell lines, miR-27a blocked AMPK and enhanced mTOR signaling, promoting aerobic glycolytic metabolism and supporting biomass production [31]. However, currently the specific effects and related mechanism of miR-27a in sepsis are not clear. In our study, the expression of miR-27a was related to the levels of GSH-Px and MDA, suggesting a potential relation with oxidative stress. Though the correlation with SOD was not significant, this result may be due to the relatively small sample size.

Early detection of sepsis would enable timely intervention and treatment, and improving outcomes. A large number of studies have shown that oxidative stress occurs during sepsis with reduced levels of SOD [20]and GSH-Px [32], and increased levels of MDA [33]. The imbalance between the ROS production and antioxidant defenses leads to sepsis [34], and oxidative markers have been measured and considered as biomarkers for sepsis in the past decades [35, 36]. Similarly, the levels of miR-27a-related MDA and GSH-Px were significantly associated with sepsis. However, the levels of MDA and GSH-Px were not significantly associated with 28-day mortality in patients with sepsis. This suggests that MDA, SOD, and GSH-Px are not potential biomarker for prediction of sepsis-related mortality. Although a series of studies have shown that alterations of oxidant/antioxidant balance is correlated with outcomes of critically ill patients [20–22].

Prior studies have reported that the acute phase proteins CRP [37] and PCT [38] are useful markers for sepsis. In the present study, the levels of CRP and PCT were increased in sepsis patients compared with non-sepsis patients. Furthermore, we demonstrated that CRP and PCT were associated with 28-day mortality in patients with sepsis, although CRP was not significantly different. However, these parameters are limited because they can be elevated in sepsis as well as other critical diseases [39–41].

Current guidelines for the treatment of sepsis recommend timely and appropriate antibiotic therapy, along with fluid resuscitation, use of vasopressors, and supportive treatment for organ failure [42]. Mortality in patients with sepsis can be further reduced by introducing goal-directed therapy and hemodynamic stabilization within the first 6 h [43]. Similarly, delays in the administration of appropriate antibiotics are associated with increased mortality in patients with sepsis [44]. Despite these efforts, the mortality rate from sepsis remains high, which suggests that a better stratification of critically ill and sepsis patients is needed to identify patients at increased risk of death [45, 46]. In this context, laboratory biomarkers and clinical scores for early diagnosis of sepsis may have special clinical significance. However, the specificity and sensitivity of current biomarkers, including CRP, PCT, and IL-6 are limited in this setting [10, 47]. More recently research has suggested that new biomarkers are need for sepsis, including the potential use of circulating miRNAs [48, 49]. In our study, we identified the value of miR-27a as a potential biomarker for diagnosis of sepsis, and explored its usefulness for predicting 28-day mortality. As a more sensitive and stable marker, altered expression of miR-27a among critically ill patients can help in the diagnosis of sepsis. Therapeutically, antioxidants could improve mitochondrial energy production and alleviate oxidative stress [27, 28], but it is still worth exploring whether better results can be achieved through the regulation of miR-27a expression.

## Conclusions

This study firstly demonstrated higher circulating miR-27a levels in patients with sepsis, which were related to a poor prognosis. There was a significant relation between miR-27a with oxidative stress, implicating that miR-27a is involved in the pathogenesis of sepsis. The results indicated that miR-27a may be as a novel biomarker in diagnosis and prognosis of sepsis, and the relations with oxidative stress may provide a promising strategy for sepsis management. However, the potential of miR-27a with respect to the diagnosis and prognosis of sepsis need to be further investigated prospective studies.

## Materials and methods

### Patients

This retrospective study was approved by the Ethics Committee of Zhejiang Provincial People’s Hospital (No. 2021QT287) and with the Helsinki Declaration of 1975, as revised in 2008. A total of 23 patients with sepsis and 25 non-sepsis patients admitted to the Emergency Intensive Care Unit (ICU) between January 2019 and January 2020 were included. A diagnosis of sepsis was based on the Third International Consensus Definitions for Sepsis and Septic Shock (Sepsis-3) [1], non-sepsis patients were those who were critically ill but did not meet the definition of sepsis. Exclusion criteria were: (1) Age < 18 years 18; (2) Systemic inflammatory response syndrome (SIRS) of non-infectious cause; (3) Receiving chronic renal replacement therapy (CRRT); (4) Recent use of antioxidant supplements before ICU admission; (5) Malignancy or HIV infection; (6) Receiving immunosuppressive treatment; (7) Body mass index (BMI) < 19 kg/m^2^.

### Data collection

A total of 10–15 ml venous blood was collected from all patients on admission, and the serum was isolated and stored at − 80 °C until analysis. Data were recorded in a standard manner for each patient and included: (1) Demographic parameters such as age and sex; (2) Clinical parameters such as comorbidities, Acute Physiology and Chronic Health Evaluation II (APACHE II) score [50], Sequential Organ Failure Assessment (SOFA) score [51], multiple organ failure, 28-day mortality, length of ICU stay and hospital stay; (3) Blood biochemical indexes including white blood cell (WBC) count, C-reactive protein (CRP), procalcitonin (PCT), superoxide dismutase (SOD), malondialdehyde (MDA), and glutathione peroxidase (GSH-Px).

### RNA extraction and reverse transcription-quantitative PCR (RT-qPCR)

Total RNA isolation was performed with a miRNeasy Mini kit (Generay, Shanghai, China), following the manufacturer’s protocol. Reverse transcription was performed using a PrimeScript™ RTRreagent kit (TaKaRa, Shiga, Japan). The qRT-PCR assays were carried out using ChamQ SYBR Color qPCR Master Mix (Vazyme, Nanjing, China) on a 7500 Fast Real-Time PCR System (Applied Biosystems, USA). U6 was used as an internal control. The primer sequences were as follows:

miR-27a Forward: 5ʹ-AGGCAGGTTCACAGTGGCTAAG-3ʹ; miR-27a Reverse: 5ʹ-GTGCAGGGTCCGAGGT-3ʹ; U6 forward: 5ʹ-CTCGCTTCGGCAGCACA-3ʹ, U6 reverse: 5ʹ-ACGCTTCACGAATTTGCG-3ʹ. The relative expression of miR-27a was reported as fold-difference relative to U6.

### Enzyme-linked immunosorbent assay (ELISA)

Serum GSH-Px, MDA, and SOD were measured by ELISA kits (JianCheng Bioengineering Institute, NanJing, China), according to the manufacturer's instructions.

### Statistical analysis

Data were presented as median (range) and count (percentage). Continuous data were compared using Mann–Whitney U test and the categorical data were compared using Fisher's exact test. Spearman’s correlation analysis was performed to evaluate the strength of relations between miR-27a and indicators of oxidative stress. Receiver operating characteristic (ROC) curve analysis was used to determine the specificity and sensitivity of miR-27a expression, CRP level, PCT level, APACHE II score, and SOFA score for diagnosis of sepsis and prediction of mortality. All data analysis was conducted using GraphPad Prism 8.0 software (GraphPad Software, La Jolla, CA, USA), and SPSS version 25.0 software (IBM Corp., New York, USA). Values of p < 0.05 were considered to indicate a statistically significant difference.

## Data Availability

The data used to support the findings of this study are included within the article.

## Abbreviations

MiR-27a: MicroRNA 27a
MDA: Malondialdehyde
GSH-Px: Glutathione peroxidase
SOD: Superoxide dismutase
TNF-α: Tumor necrosis factor alpha
IL-6: Interleukin-6
NF-κB: Nuclear factor-κB
PPARγ: Peroxisome proliferator-activated receptor γ
CRP: C-reactive protein
PCT: Procalcitonin
ROS: Eactive oxygen species
APACHE II: Acute Physiology and Chronic Health Evaluation II
SOFA: Sequential Organ Failure Assessment
CVD: Cardiovascular disease
ALI: Acute lung injury
